# Reduced neuronal population in the dorsolateral prefrontal cortex in infant macaques infected with simian immunodeficiency virus (SIV)

**DOI:** 10.1007/s13365-021-01019-2

**Published:** 2021-09-23

**Authors:** Alexandra Haddad, Brittany Voth, Janiya Brooks, Melanie Swang, Heather Carryl, Norah Algarzae, Shane Taylor, Camryn Parker, Koen K. A. Van Rompay, Kristina De Paris, Mark W. Burke

**Affiliations:** 1 Department of Physiology and Biophysics, Howard University, Washington, DC 20059, USA; 2 California National Primate Research Center, University of California Davis, Davis, CA 95616, USA; 3 Department of Microbiology and Immunology, University of North Carolina, Chapel Hill, NC 27599, USA; 4 King Saudi University, Riyadh, Riyadh, Kingdom of Saudi Arabia

**Keywords:** Pediatric HIV, Dorsolateral prefrontal cortex, Stereology, Neurodevelopment

## Abstract

Pediatric HIV infection remains a global health crisis with an estimated 150,000 new mother-to-child (MTCT) infections each year. Antiretroviral therapy (ART) has improved childhood survival, but only an estimated 53% of children worldwide have access to treatment. Adding to the health crisis is the neurological impact of HIV on the developing brain, in particular cognitive and executive function, which persists even when ART is available. Imaging studies suggest structural, connectivity, and functional alterations in perinatally HIV-infected youth. However, the paucity of histological data limits our ability to identify specific cortical regions that may underlie the clinical manifestations. Utilizing the pediatric simian immunodeficiency virus (SIV) infection model in infant macaques, we have previously shown that early-life SIV infection depletes the neuronal population in the hippocampus. Here, we expand on these previous studies to investigate the dorsolateral prefrontal cortex (dlPFC). A total of 11 ART-naïve infant rhesus macaques (*Macaca mulatta*) from previous studies were retrospectively analyzed. Infant macaques were either intravenously (IV) inoculated with highly virulent SIVmac251 at ~1 week of age and monitored for 6–10 weeks or orally challenged with SIVmac251 from week 9 of age onwards with a monitoring period of 10–23 weeks post-infection (19–34 weeks of age), and SIV-uninfected controls were euthanized at 16–17 weeks of age. Both SIV-infected groups show a significant loss of neurons along with evidence of ongoing neuronal death. Oral- and IV-infected animals showed a similar neuronal loss which was negatively correlated to chronic viremia levels as assessed by an area under the curve (AUC) analysis. The loss of dlPFC neurons may contribute to the rapid neurocognitive decline associated with pediatric HIV infection.

## Introduction

In 2015, the UNAIDS 90–90–90 initiative was launched to reduce the impact of the AIDS epidemic by having 90% of people with HIV being diagnosed, 90% on sustained antiretroviral therapy (ART), and 90% of people on ART having viral suppression by 2020 (UNAIDS-2014, 2014). However, these goals were not met in the pediatric population (aged 0–14 years) (UNAIDS 2020). Nonetheless, this past decade has seen a significant decrease in the reported worldwide number of pediatric HIV infections, with an estimated 150,000 new pediatric HIV infections in 2019, which is half the number that was reported in 2010 (UNAIDS 2020). Maternal ART and avoidance of mixed feeding practices prior to 6 months of age have been integral to the reduction of mother-to-child transmission (MTCT) (Becquet et al. 2009; Becquet et al. 2008; Rollins et al. 2013; Zash et al. 2016). Despite this advancement, children still represent 13% of the new infections in sub-Saharan Africa and 4% of global infections with the total of new pediatric infections remaining steady over the past several years (UNAIDS 2020). Globally, coverage of pediatric ART treatment is lower than that of adults, with only about 53% of HIV + children receiving treatment, leaving about 840,000 HIV + children in need of treatment (UNAIDS 2020). Although ART results in increased survival rates among HIV-infected children (Brady et al. 2010; Dowshen and D’Angelo 2011; Sohn and Hazra 2013), neurodevelopmental deficits persist (van Arnhem et al. 2013; Van Rie et al. 2007, 2008) which are compounded by the lack of ART availability (UNAIDS 2020) and challenges of ART adherence in adolescent populations (MacDonell et al. 2013; Mofenson and Cotton 2013).

Perinatally HIV-infected (pHIV) children consistently present with a higher prevalence of neurological impairment than infected adults despite controlled viremia with ART (Cohen et al. 2015; Phillips et al. 2016). Neuropsychological evaluations suggest that pHIV children display deficits in planning/reasoning, cognitive ability, motor proficiency, working memory, attention/impulsivity, IQ, and executive function (Boivin et al. 2019; Cohen et al. 2015; Nichols et al. 2016; Phillips et al. 2016; Van den Hof et al. 2019b). The neurodevelopmental sequelae of pHIV children have long-term consequences. As pHIV children transition into adolescence, planning and reasoning (Boivin et al, 2019) and executive function deficits remain (Nichols et al. 2016; Van den Hof et al. 2020). Likewise, deficits persist into adulthood with information processing speed, working memory, verbal fluency, and global cognition domains affected in pHIV adults despite being on an ART regimen (Coutifaris et al. 2020; Willen et al. 2017). Direct HIV infection of the central nervous system (CNS), systemic inflammation, and ART regimens have been implicated in pHIV neurologic impairment (Hoare et al, 2020; Musielak and Fine 2016); however, the extent of neurodevelopmental alterations and its relation to the neuropsychological manifestations remains elusive (Carryl et al. 2015; Van den Hof et al. 2019a). Imaging studies have provided evidence of CNS structural and functional differences in pHIV children, including alterations in cerebrovascular flow (Blokhuis et al. 2017; Dean et al. 2020), white matter (Ackermann et al. 2014, 2016, 2019; Donald et al. 2015; Hoare et al. 2015a, b, 2012a, b; Sarma et al. 2019, 2014), gray matter (Cohen et al. 2016; Lewis-de Los Angeles et al. 2017; Sarma et al. 2014), and decreased total brain volumes (Dean et al. 2020). Altered functional connectivity has been reported in the left middle temporal gyrus, postcentral gyrus, and middle frontal gyrus, potentially affecting auditory and visual coordination, sensory network, and cognitive networks, respectively (Yadav et al. 2017, 2018). Imaging and neuropsychological assessments indicate that the prefrontal cortical network, at least in part, is involved in the cognitive deficits in pHIV (Goldman-Rakic. 1995a, 1996; Herting et al. 2015; Levy and Goldman-Rakic 1999; McCarthy et al. 1996; Rajkowska and Goldman-Rakic. 1995a, b).

Although imaging data suggests an altered prefrontal cortical network, these studies lack the sensitivity to identify alterations at the cellular level and characterize the pathophysiology of pHIV. The scarce pathology reports suggest cortical apoptosis (Gelbard et al. 1995) and an active role of astrocytes in the neuropathogenesis in pHIV infection (Blumberg et al. 1994; Saito et al. 1994; Tornatore et al. 1994; Trillo-Pazos et al. 2003). One of the main obstacles in pHIV research is sample access necessitating animal model systems to investigate the pathogenesis of HIV in the developing brain (Carryl et al. 2015; McLaurin et al. 2017a, b, 2018a, b, 2020, 2017c; Moran et al. 2019). The HIV-1 transgenic rat (HIVTg), which expresses 7 of the 9 HIV-1 proteins throughout its lifespan, including the developmental period, has shown deficits in temporal processing (Moran et al. 2013), attention, and executive function components of inhibition and flexibility (Moran et al. 2014a). Further supporting the vulnerability of the frontal cortex, HIVTg animals display altered morphology of pyramidal neuronal dendritic spines of layers II–III in the medial prefrontal cortex (McLaurin et al. 2018b). While rodent models have primarily focused on the effects of viral proteins on the developing brain (McLaurin et al, 2017a, b, 2018a, b; Moran et al. 2014b), the neuropathogenic effects of pHIV infection are not recapitulated in these models (Carryl et al.2015).

The pediatric simian immunodeficiency virus (SIV) model complements rodent models since macaques have a similar developmental pattern (both immune and neurodevelopment) to humans. Additionally, SIV and HIV-1 have similar pathogenesis, routes of transmission, immune suppression, and disease progression (Abel 2009; Carryl et al. 2015; Clancy et al. 2001; Nowakowski and Rakic 1981). Furthermore, MTCT can occur by the same routes in rhesus macaques and humans, and we have recently shown that within 96 h of oral SIVmac251 administration, viral RNA and DNA can be detected in the cerebral cortex (Abel 2009; Amedee et al. 2018). Subjects that either received intravenous SIVmac251 within 1 week of age or were orally challenged at 9 weeks of age displayed a significant loss of neurons throughout the CA subfields (CA1–3) (Carryl et al. 2017; Curtis et al. 2014) along with reduced immature neuronal population in the dentate gyrus (Curtis et al. 2014). While the hippocampus is a key component of the cognitive network (Friedman and Goldman-Rakic. 1988; Lisman et al. 2017), deficits within this area cannot alone account for the cognitive and executive deficits. Available clinical evidence (Boivin et al. 2019; Cohen et al. 2015; Nichols et al. 2016; Phillips et al. 2016; Van den Hof et al. 2019a; Van den Hof et al. 2019b) suggests involvement of the prefrontal cortex, in particular the dorsolateral prefrontal cortex (dlPFC) which lies within the superior and middle frontal gyri in humans and the principle sulcus in non-human primates (Friedman and Goldman-Rakic, 1988; Goldman-Rakic 1995a, 1996; Levy and Goldman-Rakic 1999; McCarthy et al. 1996; Petrides and Pandya 1999; Rajkowska and Goldman-Rakic 1995a, b); however, this area is relatively unexplored in models of pHIV. Here, we identify the effects of pediatric SIV infection on the neuronal population in the dlPFC.

## Methods

### Subjects and procedures

Infant macaques (*Macacca mulatta*) born to SIV-naïve dams were nursery-reared at the California National Primate Research Center (CNPRC) in accordance with the American Association for Accreditation of Laboratory Animal Care Standards. Subjects in the current study were part of previously conducted studies (Jensen et al. 2017, 2016, 2013, 2012) with all procedures approved by the University of California at Davis Institutional Animal Care and Use Committee. Briefly, a total of 12 infant rhesus macaques were included in this study consisting of (1) an intravenously SIVmac251-inoculated neonatal group (1 inoculation with 1000 median tissue culture infectious dose (TCID_50_) at ~1 week of age; IV group, *n* = 3) with a monitoring time of 6–10 weeks, (2) an orally SIVmac251-inoculated group (PO group, *n* = 5) that received weekly low-dose SIVmac251 regimen (5000 TCID_50_) starting around 9 weeks of age until infected with a monitoring time of 10–23 weeks post-infection, and (3) an SIV-naïve control group (*n* = 4) that was euthanized between 16 and 17 weeks of age (Table 1). SIVmac251 was obtained from the Analytical Resource Core at the CNPRC (Abel 2009), and plasma viral loads were determined from weekly blood samples and quantified by real-time reverse transcription polymerase chain reaction (Cline et al. 2005; Jensen et al. 2016). All animal procedures were performed under ketamine-HCl anesthesia (10 mg/kg i.m.; Parke-Davis, Morris Plains, NC). Sample collections, sample processing, and euthanasia were performed as previously described (Jensen et al. 2016, 2012).

### Histology

Immediately following euthanasia, brains were extracted, fixed in 10% formalin, blocked into 1-cm slabs, cryoprotected in graded sucrose (10–30%), frozen at −65 °C in isopentane, and stored at −80 °C until further processing (Carryl et al, 2017; Curtis et al. 2014). Blocks of tissue were then systematically sectioned in the coronal plane (50 μm) at −20 °C in a Microm cryostat in 10 parallel series. The first series of each block was Nissl-stained with cresyl-violet for design-based stereology. The remaining series of tissue were placed in antigen preserve (50% ethylene glycol, 1% polyvinal pyrrolodone in phosphate buffer pH = 7.4-PBS), and stored in numbered tubes at −20 °C for immunohistochemistry as part of our brain bank (Burke et al. 2009b; Carryl et al. 2017; Curtis et al. 2014).

### Design-based stereology

**Fig. 1:**
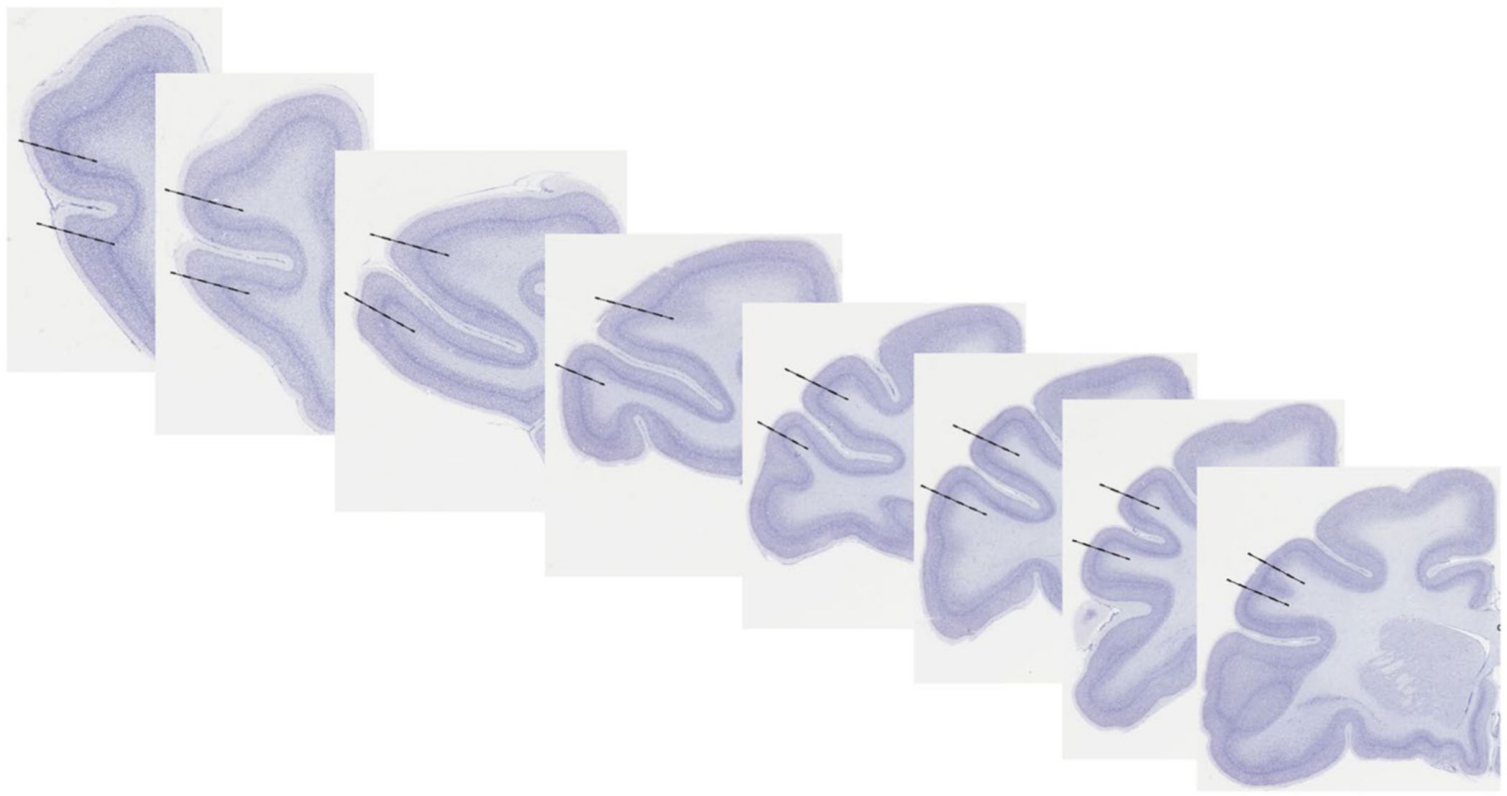
The rostro-caudal extent area 46/9dv which includes the principle sulcus extending onto the “lips” of the sulcus (Paxinos et al. 2008; Petrides and Pandya 1999; Petrides et al. 2012). The dotted lines demonstrate approximate extent of the counting region. Images are modified from www.BrainMaps.org

Quantification of the neuronal population in the dlPFC was achieved using design-based stereology with the optical fractionator method (Burke et al. 2009a; Carryl et al. 2017; Curtis et al. 2014). The dlPFC was delineated on the basis of cytoarchitecture and fiduciary landmarks to include areas 46, 9/46d, and 9/46v (Fig. 1) (Paxinos et al, 2008; Petrides and Pandya, 1999; Petrides et al, 2012). Briefly, the principal sulcus was used as a fiduciary landmark as area 46 occupies the banks of the sulcus and areas 9/46d (dorsal) and 9/46v (ventral) are located on the lips of the sulcus. Cytoarchitecturally, these regions have similar, well-defined layers II, III, IV, and V (Petrides and Pandya 1999) which were used to delineate from adjacent regions. Equidistant sections were sampled throughout the entire rostral-caudal length of this area, and a minimum of two observers were used to delineate each section to ensure consistency between sections and subjects. Sampling parameters for design-based stereology are similar to previously described methods (Carryl et al. 2017; Curtis et al. 2014) where the topography was performed using a 5 × objective and an *x*–*y* sampling grid (1000 μm^2^) with superimposed counting frames (16,000 μm^3^) generated through the Stereologer System (Stereology Resource Center, Inc., Tampa, FL, USA). All counting was performed using a plan fluor oil immersion 63 × objective (N.A. 1.4), and every 40th section was counted with a random starting point within the rostral 2000 μm of the dlPFC. The Cavalieri estimator was used to determine reference volume of the dlPFC (West and Gundersen 1990). Mean cell volume (MCV) of neurons was simultaneously determined using the nuclear rotator parameter of the Stereologer system, so that neurons that were counted in the total population estimate were also measured as part of the MCV. The total estimation of cell numbers (*N*) were calculated by the following equation: *N* = ssf^−1^ × asf^−1^ × tsf^−1^ × ΣQ^−^. Where ssf is the section sampling fraction, asf is the sampling fraction, tsf is the thickness sampling fraction (where the measured thickness of the tissue is divided by the disector height), and ΣQ^−^ is the total number of neurons (defined as having a visible centrally located nucleoli and clearly defined cytoplasm) counted within the dissector (Joelving et al. 2006). MCV was calculated as MCV = mean *l*^*3*^ × 4π/3, where *l* is the length of the line proportional to the area of the object (Mouton et al. 1994).

### Immunohistochemistry

Standard immunohistochemical techniques were followed for p27 immunohistochemistry, a core protein of SIVmac251 (Higgins et al. 1992). Briefly, matched sections from the dlPFC were removed from the brain bank, washed 3 times in PBS to remove residual antigen preserve. Free-floating sections were then incubated for 20 min in a 3% hydrogen peroxide and 20% methanol solution in PBS to quench endogenous peroxidase activity. Sections were then washed in PBS, blocked in 3% normal horse serum, and then incubated with mouse anti-SIVmac251 p27 monoclonal antibody (1:200 dilution; NIH AIDS Reagent Program catalog #1610) overnight at 4 °C. Tissues were then washed in PBS and incubated in biotinylated multilink secondary antibody (BioGenex, #LP000-ULE) at room temperature for 20 min. Following an additional set of washes in PBS, the sections were then incubated in a streptavidin–horseradish peroxidase solution (BioGenex, #LP000-ULE) at room temperature for 20 min. Sections were visualized with diaminobenzidine (DAB #4418, Sigma, St. Louis, MO, USA), mounted on gelatinized slides, dehydrated in graded alcohols (50–100%), cleared in xylenes, and coverslipped with permount mounting media (Fisher Scientific, #SP15).

### Statistical analysis

Due to the small group sizes, statistical differences were determined only between two groups at a time applying both one-tailed (Carryl et al. 2017) and two-tailed non-parametric Mann–Whitney *U* test of significance using the InStat3 program (La Jolla, California, CA, USA). The coefficient of variation (CV = SD / mean) was calculated for neuronal population and regional and cell volumes. Coefficients of error (CE) were calculated for total number of neurons to assess the reliability of measurements with average CE being calculated as √ meanCE ^2^. Correlations between neuronal population or neuronal size and SIV viremia were assessed by Spearman rank test using GraphPad Prism version 9.1 (GraphPad, La Jolla, CA).

## Results

Here, we report a comparison of the dlPFC neuronal population, neuronal size, and volume in subjects infected IV with SIVmac251 shortly after birth (within 1 week), PO during the early infancy stage (9–17 weeks of age), and control subjects (Table 1). A set of 8–11 sections spanning the rostral-caudal extent of the dlPFC were sampled with an 1000-μm^2^ *x–y* grid resulting in an average of 277 ± 52 disectors per subject (control 312.25 ± 33.08; IV 230.67 ± 43.25; and PO 277.55 ± 53.33). The average TSF was similar between groups (control 17.42 ± 1.72 μm; IV 16.94 ± 0.68 μm; and PO 15.18 ± 0.77 μm).

**Fig. 2:**
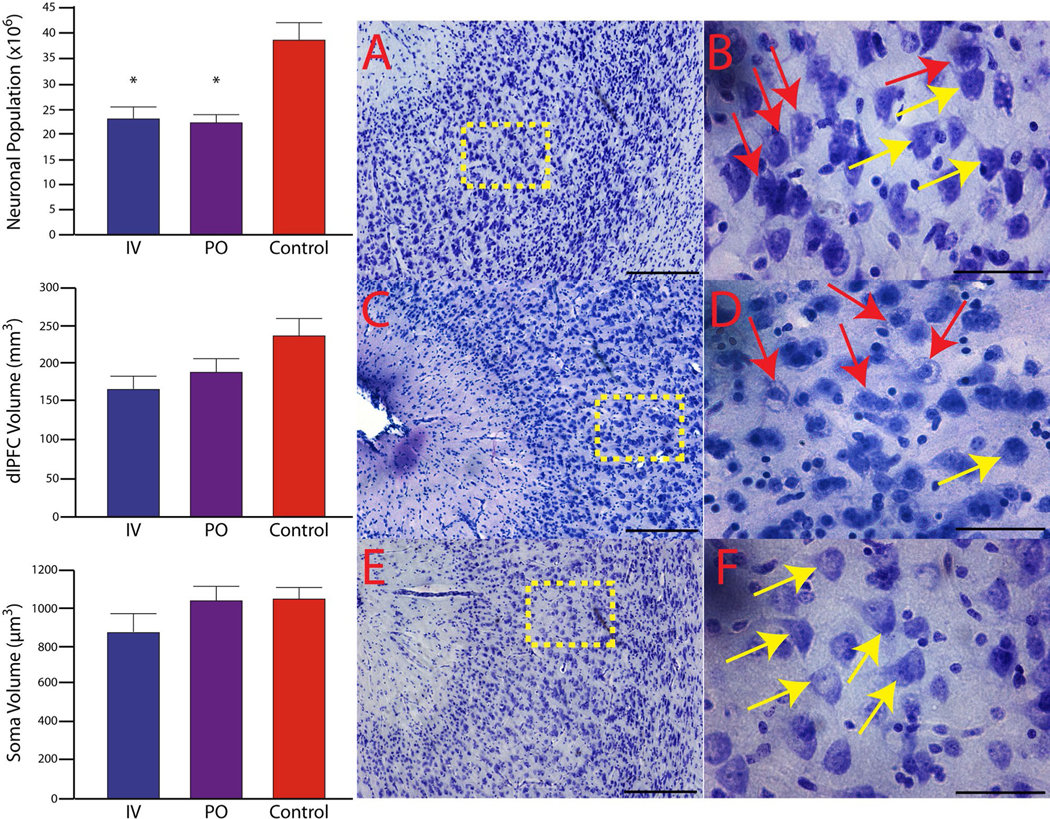
There were significant neuronal reductions within the dlPFC of SIV-infected subjects compared to the control group. Overall dlPFC regional volume and neuronal soma volume were not different between groups. *p < 0.05, one-tailed IV vs control and PO; two-tailed PO vs control. In addition to the reduced neuronal populations, there is evidence of ongoing neuronal death in both the IV- (**A**, **B**) and PO-infected (**C**, **D**) groups centered around the genu and extending into the dorsal and ventral banks of the principle sulcus and mostly absent in control (**E**, **F**) subjects. Red arrows in **B** and **D** indicate neurons with pyknotic nuclei and vacuoles indicative of neurons in the process of dying. Yellow arrows in the IV- (**B**), PO (**D**), and control (**F**) groups indicate healthy-looking neurons. Yellow dashed boxes in panels **A**, **C,** and **E** indicate approximate area where higher-magnification images were taken. Images **A**, **C**, and **E** were taken at 10 ×, scale bar = 250 μm, and **B**, **D**, and **F** were taken at 63 ×, scale bar = 50 μm

**Fig. 3:**
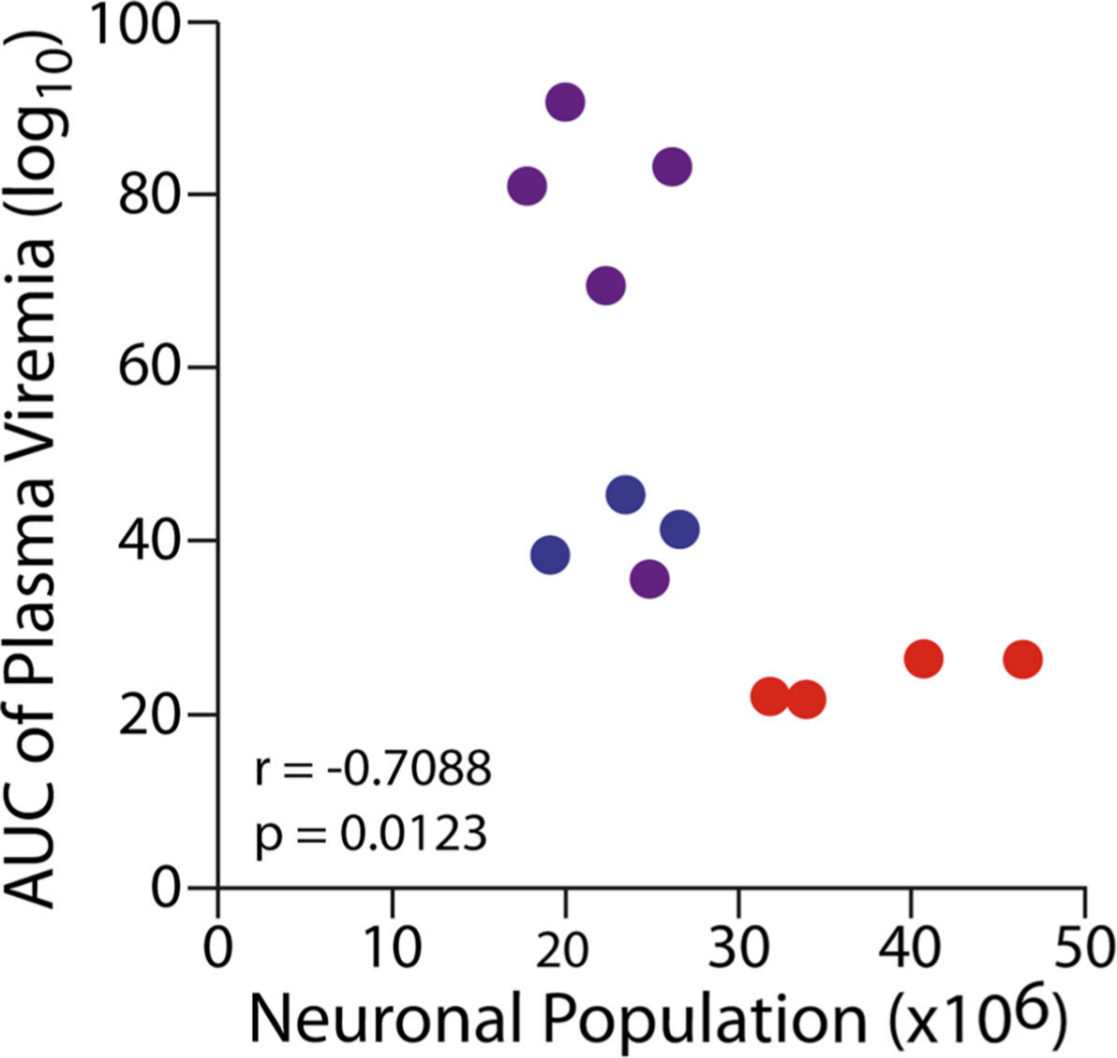
AUC analysis indicates a significant negative correlation between plasma viremia and neuronal population in the dlPFC

Pediatric SIV infection resulted in a decrease in the total neuronal population of the dlPFC (KW = 7.477, *p* = 0.0057; Fig. 2). Compared to control subjects (38.23 ± 3.35 million neurons; CV = 0.154), IV SIV-infected subjects had a 49.5% neuronal reduction (23.06 ± 2.18 million neurons; CV = 0.175; *p* = 0.0286), and PO SIV-infected subjects had a 52.8% neuronal reduction (22.26 ± 1.54 million neurons; CV = 0.154; *p* = 0.016). There was no statistical difference in neuronal populations between IV and PO SIV-infected subjects (*p* = 0.393). The average CE was below 0.05. The decrease in neuronal populations in the dlPFC was correlated with viremia AUC (Fig. 3).

**Fig. 4:**
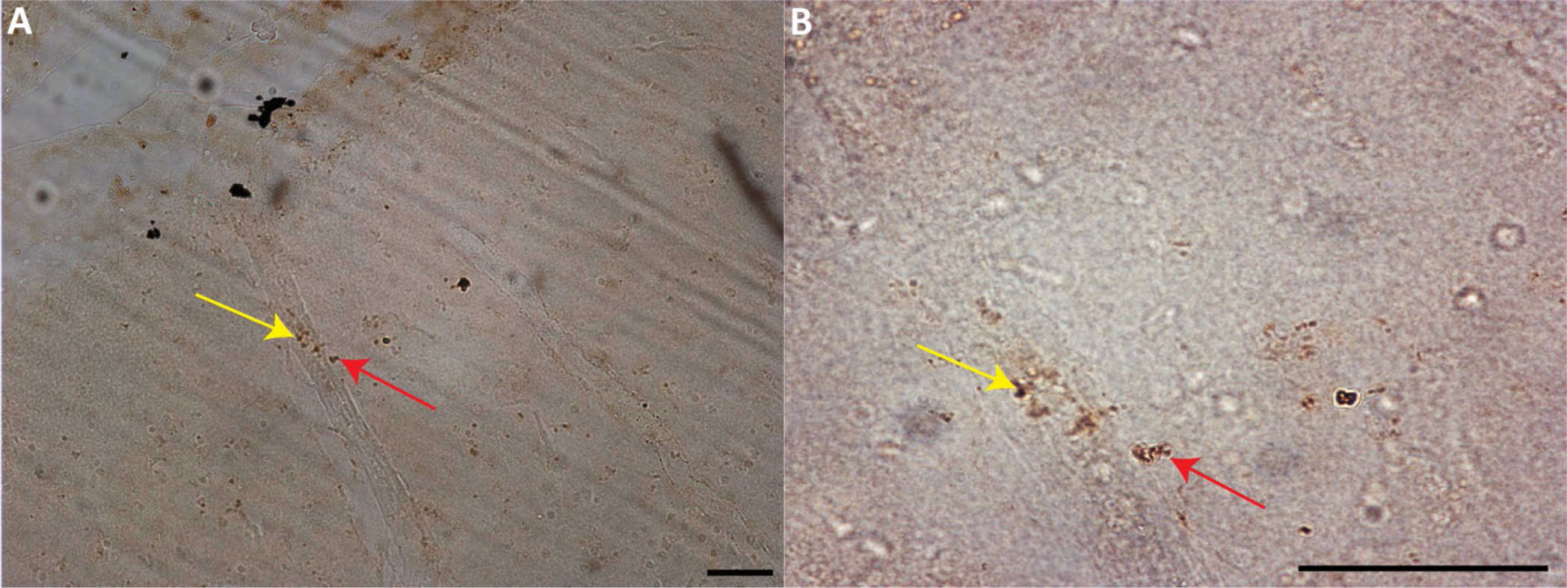
Immunohistochemical analysis demonstrates p27-positive staining throughout the dlPFC and adjacent pia matter. In panel **A**, the yellow arrow indicates a blood vessel with p27-positive staining inside the vessel and the red arrow p-27 outside the vessel within the brain parenchyma. In panel **B**, the red arrow shows the perivascular p27 location. Panel **A** was taken at 20 ×, and **B** was taken at 63 ×, scale bar = 50 μm

The estimated volume of the dlPFC was similar between groups (control 237.4 ± 2.1 mm^3^, CV = 0.1806, and average CE = 0.006; IV 165.8 ± 1.7 mm^3^, CV = 0.1752, and average CE = 0.005; PO 189.4 ± 1.7 mm^3^, CV = 0.1975 and average CE = 0.007; KW = 3.958, *p* = 0.093; Fig. 2). To determine average neuronal soma volume, a minimum of 400 neurons per subject were sampled. Neuronal soma volume did not differ between groups (KW = 1.985, *p* = 0.269; Fig. 2). The average CE was below 0.05 for both regional and neuronal volumes. Consistent with similar neuronal size and volumes between the groups, there was no correlation between viremia and neuronal size or volume. A consistent observation with each of the SIV + subjects was numerous cytoplasmic inclusions of neurons within the genu, dorsal, and ventral banks of the principle sulcus indicative of neurons in the process of dying (Fig. 2). Neurons within this region display pyknotic nuclei with adjacent clear spaces with basophilic structures outlining clear vacuoles (Christopher et al. 2017; Greaves 2007; Little et al. 1974). Within this same region, there was also positive immunostaining for p27 (Fig. 4).

## Discussion

We have previously reported a significant reduction of hippocampal neuronal populations in both PO and IV SIV-infected subjects (Carryl et al. 2017; Curtis et al. 2014). The hippocampus is critical in learning and memory, but it is a particularly vulnerable region to intrusions throughout the lifespan (Bartsch and Wulff 2015). Hippocampal neurons are susceptible to Tat-mediated excitotoxicity (Campbell et al. 2011) and have been shown to be a vulnerable region in both adult and developmental models of HIV toxicity (Carryl et al. 2015). Given its susceptibility, the hippocampus was a logical starting point to assess the neurological impact of SIV on the developing brain (Carryl et al. 2015, 2017), although clinical evidence suggests disruptions throughout the cognitive circuitry (Boivin et al. 2018; Boivin et al. 2019; Cohen et al. 2015; Nichols et al. 2015; Nichols et al. 2016; Phillips et al. 2016; Van den Hof et al. 2019b; Van den Hof et al. 2020). Here, we expanded on our previous results to show that the dlPFC is vulnerable to the deleterious effects of post-natal SIV-infection.

The developing brain undergoes rapid development and reorganization beginning in the early fetal period through the second year of life (Kostovic and Rakic, 1980) with the pattern and tempo being remarkably similar between human and non-human primates (Huttenlocher 1990; Huttenlocher and Dabholkar 1997; Huttenlocher et al. 1982; Kostovic et al. 2019; Rakic 1978). This is especially true for the dlPFC which is a functionally advanced region of the brain that is responsible for attention, planning, executive function, decision-making, and mediating working memory (Beveridge et al. 2014; Fuster 2002; Goldman-Rakic 1987; Levy and Goldman-Rakic 1999). The dlPFC is one of the final regions of the cortex to fully develop, both functionally and structurally, and it continues developing throughout young adulthood (Beveridge et al. 2014). In both species, the period of rapid neurogenesis and neuronal migration is concentrated during the first two trimesters (Kostovic et al. 2019; Rakic 1978). The third trimester through early infancy marks a period of rapid maturation of the dlPFC whereby disruption of the developmental process may result in long-term neurological consequences (Spencer-Smith and Anderson 2009). A critical component of cortical network and functional maturation is synaptogenesis. Within the human dlPFC, the rapid phase of synaptogenesis begins during the third trimester of pregnancy continuing through the first 15 postnatal months (Huttenlocher and Dabholkar 1997). There is a plateau of synaptic densities from around 1–10 years old, followed by synaptic elimination during late childhood and adolescence. The time course of synaptogenesis resembles dendritic development and myelination. Similarly, in non-human primates, there is a rapid synaptogenesis phase in the dlPFC beginning during the last 2 months of pregnancy lasting through the first 2 postnatal months. After that, there is a constant synaptic density lasting through 3 years of age, followed by a steady decline from 3 to 20 years of age (Bourgeois et al. 1994).

In the current study, subjects were infected either during the neonatal or early infancy period concurrent with rapid synaptogenesis and maturation resulting in a significant reduction in neurons in the dlPFC. We found evidence of neurons with cytoplasmic inclusions and vacuoles indicative of actively dying cells confined primarily to the genu and extending into the dorsal and ventral banks of the principle sulcus. Although it is known that HIV and SIV induce neuronal death (Beck et al. 2015; Kaul 2008; Kaul et al. 2001), the clustering of apparently dying neurons within the genu region in close proximity to p27 immunoreactivity, was not observed on the upper or lower lips of the principle sulcus or in the hippocampus (Carryl et al. 2017), indicating that this region may be particularly vulnerable. Apoptosis and inflammation along with autophagy are probable causes of HIV/SIV-associated neuronal death (Saylor et al. 2016; Zhou et al. 2011). During apoptosis, cells initiate an innate suicide program that results in self-destruction either through phagocyte recruitment and/or their enveloping (Perez-Garijo et al. 2013). Apoptosis leads to altered morphology of a cell, with the cytoplasm appearing denser and more contained. One of the morphological indicators of apoptosis is pyknosis—the process of chromatin condensation (Elmore 2007; Voss and Strasser 2020), which we observed within the genu of the principle sulcus corresponding to area 46 of the dlPFC. The HIV envelop glycoprotein (gp120) and transactivator of transcription (Tat) have been shown to directly induce neuronal apoptosis (Bagashev and Sawaya 2013; Kaul 2008) and may contribute to the neuronal loss in the dlPFC. Additionally, microglia and astrocytes (which are suspected of harboring the latent HIV reservoir) are capable of productive HIV infection and play a significant role in the indirect inflammatory neurotoxic cascade of HIV (Bozzelli et al. 2019; Li et al. 2020; Mocchetti et al. 2013; Pandey and Seth 2019). However, pediatric SIV infection may affect the dlPFC beyond neuronal death. Given that this developmental time point corresponds to rapid synaptogenesis and dendritic lengthening, it is conceivable that this process is compromised. In addition to inducing neuronal death, HIV has also been shown to affect synaptic levels and is implicated as a mechanism of cognitive dysfunction in adult HIV/SIV (Gupta et al. 2010; Wenzel et al. 2019). In fact, the HIV protein gp120 has been shown to reduce dendritic length (Avdoshina et al. 2020) and induce neurite pruning (Speidell et al. 2019). Pediatric HIV may disrupt the neuron–microglia interaction, in a non-inflammatory manner, that affects the normal role that microglia play in selective synapse elimination which is critical for maturation and network development (Paolicelli et al. 2011; Paolicelli and Ferretti 2017; Paolicelli and Gross 2011). During development, neurons upregulate the release of CXCL1 (fractalkine) that bind to the receptor CXCR1 expressed on microglia, leading to synaptic pruning and strengthening of the neuronal electrophysiological properties (Paolicelli and Ferretti 2017). However, the HIV protein Tat reduces CX3CR1 expression compromising the ability of microglia to guide the maturation process (Duan et al. 2014) potentially leading to an excess of weak synapses affecting the foundation of the developing neural network (Paolicelli et al. 2011; Paolicelli and Ferretti 2017; Paolicelli and Gross 2011). Likewise, astrocytes actively promote synaptogenesis, post-synaptic receptor clustering, and dendritic arborization (Li et al. 2020; Shan et al. 2021). Astrocyte dysfunction, as a result of HIV infection, is associated with compromised blood–brain barrier integrity, neuronal survival as well affecting synapses through a Tat-mediated release of extracellular vesicle microRNA-7 leading to reduced synaptic densities by downregulating neuroligin-2 in neurons (Hu et al. 2020; Ton and Xiong 2013). Altered neuronal morphology and synaptogenesis and the roles of microglia and astrocytes during this critical period warrant further investigation within this model as it could further affect cortical maturation with long-lasting consequences especially in cognitive and executive domains (Spencer-Smith and Anderson 2009). The lack of differences in neuronal populations between the IV- or PO-infected groups further suggests that post-natal period is vulnerable period for the neurotoxic effects of HIV infection. This conclusion is further suggested by the finding that even the animal with the lowest plasma viral load at euthanasia displayed neuronal loss. To account for the overall SIV replication, we calculated the AUC of viremia over time. AUC viremia was negatively correlated with neuronal populations in the dlPFC, whereas, in the same subjects, the hippocampal neuronal loss was not associated with plasma viral load (Carryl et al. 2017). The current study suggests a regional variation that may correlate with clinical data suggesting poorer neurocognitive outcomes with higher viral load in pHIV within the first 3 years of life (Weber et al. 2017). Future studies need to determine if SIV present in the brain or SIV-induced inflammation is the primary driver of neuronal death.

The role of the dlPFC and its relationship to the deficits seen in pediatric HIV have its roots in its functional connectivity. The dlPFC which encompasses the superior and middle frontal gyri (areas 9 and 46) in humans and the principle sulcus including the dorsal and ventral banks in non-human primates has extensive cortico-cortical connections (Petrides and Pandya 1999). Tracing studies in non-human primates shows extensive connections with multimodal, motor, and paralimbic regions. Within the motor domain, the dlPFC is reciprocally connected with the supplementary motor area and motor regions of the cingulate cortex to influence the initiation and execution of skilled movements (Bates and Goldman-Rakic 1993; Lu et al. 1994). Multimodal temporal cortical areas along with paralimbic cortical areas (cingulate, retrosplenial, and rostral temporal cortex) share reciprocal connections with the dlPFC providing a functional link between the dlPFC and hippocampus along with playing a role in spatial cognition (Mitchell et al. 2018; Morris et al. 1999; Petrides and Pandya 1999).

Well-documented studies have demonstrated the significant roles of various brain regions in executive and cognitive function such as the hippocampus and dlPFC. Executive function can be described as the assemblance of cognitive processes that are needed to successfully commence, track, and manage actions and thoughts (Walker and Brown 2018). In child development, executive function plays a critical role in various areas such as moral and communicative behavior, and social cognition (Kochanska et al. 1997; Moriguchi et al. 2010, 2008). Executive function can be divided into three main compartments: shifting, inhibition, and working memory (Moriguchi and Hiraki 2013). The dlPFC has consistently been identified as a region that plays a vital role in visuospatial working memory in adolescents, adults, and non-human primates (Braver et al, 1997; Casey et al. 2005; Goldman-Rakic 1995a, b, 1996). The consistently reported neuropsychological findings of deficits of cognition and executive function in pHIV-infected children (Boivin et al. 2018; Boivin et al. 2019; Boivin et al. 1995; Cohen et al. 2015; Coutifaris et al. 2020; Nichols et al. 2015; Phillips et al. 2016; Van den Hof et al. 2019b; Van den Hof et al. 2020; Willen et al. 2017) strongly implicate dysfunction of the dlPFC network. Data presented here provide the anatomical basis of neuronal loss that may, in part, underlie the cognitive and executive dysfunction in pHIV infection. It should be noted, however, that the subjects in this study are ART-naïve and while most HIV + children in resource-rich countries have access to ART, neurocognitive deficits persist (van Arnhem et al. 2013). Thus, future studies investigating the effects of ART on brain development of SIV-infected infant macaques are warranted.

## Conclusion

The findings of this study are significant due to the clinical implications in pHIV-infected children as they move through adolescence and adulthood. The long-term neuropsychological deficits in pHIV may impact academic performance and self-management permeating all facets of life, necessitating early intervention strategies aimed at minimizing the neurodevelopmental impact (Mofenson and Cotton 2013; Sirois et al. 2016). Here, we describe neuronal loss in the dlPFC which may, in part, underlie the executive dysfunction reported in pHIV infection. Since executive and cognitive function relies on multiple regions as part of a network, our data do not preclude vulnerabilities in regions outside of the hippocampus and dlPFC. Given that psychiatric disorders are reported in vertically infected adolescents, it is quite possible that deficits in limbic-related regions begin early in the pathogenesis of pHIV infection. pHIV adolescents are more susceptible to neurocognitive deficits (Bisiacchi et al. 2000; Foster et al. 2012; Jeremy et al. 2005; Kapetanovic et al. 2010; Laughton et al. 2013; Smith et al. 2006; Walker et al. 2013) and depression (Gadow et al. 2012) than HIV + adults that contribute to lower self-esteem and poor adherence to medication (Hazra et al. 2010; MacDonell et al. 2013; Sohn and Hazra. 2013). Youth have the most to gain from an intervention that would improve self-management of HIV, because they have the longest life-years to live with the disease and are at high risk for transmission. Neurocognitive impairment in pHIV infection is associated with a greater risk for disease progression and poorer morbidity even in the advent of antiretroviral therapy (Pearson et al. 2000).

## Figures and Tables

**Table 1:** Comparison of control, IV-, and PO-infected groups

Group	Subject	Gender	Age of SIV infection	Age at euthanasia	Total infection period	Plasma SIV RNA (copies/mL)^a^

IV-infected	RM1	M	1 week	10 weeks	9 weeks	160,000,000
IV-infected	RM2	F	1 week	7 weeks	6 weeks	240,000,000
IV-infected	RM3	F	1 week	10 weeks	10 weeks	650,000,000
PO-infected	RM4	F	9 weeks	21 weeks	12 weeks	5,800,000
PO-infected	RM5	F	17 weeks	27 weeks	10 weeks	6,400,000
PO-infected	RM6	F	10 weeks	22 weeks	12 weeks	380,000
PO-infected	RM7	F	13 weeks	25 weeks	12 weeks	46,000,000
PO-infected	RM8	M	9 weeks	21 weeks	12 weeks	71,000,000
Control	RM9	F	N/A	16 weeks	N/A	N/A
Control	RM10	M	N/A	16 weeks	N/A	N/A
Control	RM11	F	N/A	15 weeks	N/A	N/A
Control	RM12	F	N/A	16 weeks	N/A	N/A

a Plasma SIV RNA levels were taken at the time of euthanasia. The PO group was orally exposed to SIVmac251 starting at 9 weeks of age once weekly until infection was verified (Jensen et al. 2017, 2016, 2013)
