# Correction to: Reduced neuronal population in the dorsolateral prefrontal cortex in infant macaques infected with simian immunodeficiency virus (SIV)

**DOI:** 10.1007/s13365-023-01185-5

**Published:** 2024-02-01

**Authors:** Alexandra Haddad, Brittany Voth, Janiya Brooks, Melanie Swang, Heather Carryl, Norah Algarzae, Shane Taylor, Camryn Parker, Koen K. A. Van Rompay, Kristina De Paris, Mark W. Burke

**Affiliations:** 1 Department of Physiology and Biophysics, Howard University, Washington, DC 20059 USA; 2 California National Primate Research Center, University of California Davis, Davis, CA 95616 USA; 3 Department of Microbiology and Immunology, University of North Carolina, Chapel Hill, NC 27599 USA; 4 Department of Physiology, College of Medicine, King Saud University, Riyadh, 11149 Saudi Arabia

**Correction to: Journal of NeuroVirology (2021) 27:923-935**

10.1007/s13365-021-01019-2

There is a typographical error in author affiliation #4. The correct affiliation is: Department of Physiology, College of Medicine, King Saud University, Riyadh 11149, Saudi Arabia

